# Clustering and switching analysis of verb fluency in individuals with Alzheimer’s disease

**DOI:** 10.1590/2317-1782/20232021179en

**Published:** 2023-04-17

**Authors:** Bárbara Costa Beber, Franceia Veiga Liedtke, Felipe Schroeder de Oliveira, Lucas Müller-Silveira, Emily Viega Alves, Márcia Lorena Fagundes Chaves, Jerusa Fumagalli de Salles

**Affiliations:** 1 Departamento de Fonoaudiologia, Universidade Federal de Ciências da Saúde de Porto Alegre - UFCSPA - Porto Alegre (RS), Brasil.; 2 Programa de Pós-graduação em Ciências da Reabilitação, Universidade Federal de Ciências da Saúde de Porto Alegre - UFCSPA - Porto Alegre (RS), Brasil.; 3 Programa de Pós-graduação em Psicologia, Universidade Federal do Rio Grande do Sul - UFRGS - Porto Alegre (RS), Brasil.; 4 Curso de Psicologia, Universidade Franciscana - UFN - Santa Maria (RS), Brasil.; 5 Curso de Fonoaudiologia, Universidade Federal de Ciências da Saúde de Porto Alegre - UFCSPA - Porto Alegre (RS), Brasil.; 6 Departamento de Medicina Interna, Programa de Pós-graduação em Medicina - Ciências Médicas, Universidade Federal do Rio Grande do Sul - UFRGS - Porto Alegre (RS), Brasil.; 7 Departamento de Psicologia do Desenvolvimento e da Personalidade, Universidade Federal do Rio Grande do Sul - UFRGS - Porto Alegre (RS), Brasil.

**Keywords:** Alzheimer’s Disease, Cognition, Language, Neuropsychology, Dementia

## Abstract

**Purpose:**

To investigate verb fluency performance in individuals with Alzheimer’s disease compared with healthy older adults by analyzing total correct responses, number of clusters, average cluster size, and number of switches.

**Methods:**

This is a case-control study of 39 healthy older adults and 29 older adults with a diagnosis of Alzheimer’s disease. Verb fluency performance was analyzed in terms of total number of correct verbs retrieved, number of clusters, average cluster size, and number of switches. To obtain the study outcomes, we previously conducted a procedure for categorization of the verbs that would compose the clusters. The classification of verbs was adapted for this study, including assessment by raters and analysis of inter-rater reliability.

**Results:**

Individuals with Alzheimer’s disease showed significantly poorer performance than healthy controls in the number of switches and total number of correct verbs retrieved. The two groups did not differ significantly in the other measures.

**Conclusion:**

In this study, individuals with Alzheimer’s disease showed impaired verb fluency, characterized by a reduced number of verbs retrieved and fewer transitions between verb categories. The findings suggest that, in Alzheimer’s disease, verb fluency is more sensitive to cognitive deficits resulting from executive dysfunction than from semantic disruption.

## INTRODUCTION

Verbal fluency is a neuropsychological assessment in which participants should produce, usually within one minute, as many words as possible from a given cue. According to word production criteria, verbal fluency can be divided into semantic or category fluency (generation of words from a specific semantic category, such as animals or fruits), phonemic or letter fluency (generation of words beginning with a specified letter, where the letters “f”, “a”, and “s” are the most used), action or verb fluency (generation of verbs or words that represent “things that people do”), and free fluency^(1-3)^. Overall, verbal fluency tasks require cognitive skills in language, semantic memory, executive function, and working memory^(4)^. However, each task type involves different cognitive demands and may recruit certain areas of the brain according to the criteria used for word production^(5,6)^. High levels of education and general cognitive functioning are associated with better performance in verbal fluency tasks^(5,6)^.

Verb fluency is more complex than traditional noun fluency tasks, as verbs have more inflections and more syntactic relationships with other words in a sentence than nouns^(7)^. Additionally, when retrieved, nouns and verbs activate different areas of the brain. Retrieval of common and proper nouns is primarily mediated by posterior and anterior temporal regions, respectively, whereas verb retrieval is primarily mediated by frontal regions, highlighting the potential utility of verb fluency as an indicator of executive function abilities^(8,9)^. Verb fluency has therefore been suggested as a marker of frontostriatal dysfunction given its sensitivity to the integrity of these brain networks and as a novel measure of executive function and linguistic skills^(7,9)^.

Verbal fluency tasks are widely used as part of cognitive assessment in individuals with neurodegenerative diseases. Verbal fluency impairment has been described in Alzheimer’s disease (AD), especially impairment in semantic fluency, which is more commonly affected than phonemic fluency^(10-12)^. These characteristics are related to the predominant involvement of left temporal lobe regions linked to semantic memory processing^(11,13,14)^. Conversely, studies investigating verb fluency in AD reported that people with AD showed poorer performance than their healthy peers^(12,15)^ and those with mild cognitive impairment^(16)^. This task was also indicative of the conversion from cognitive health to mild cognitive impairment^(17)^. However, it remains unclear whether the cognitive verb fluency deficits observed in people with AD are either executive or semantic in nature, or both. To this end, methods that analyze not only the total number of verbs retrieved but also their characteristics and forms of retrieval (clustering and switching) may be particularly useful.

Traditionally, performance in verbal fluency tasks, including verb fluency, is assessed by the total score of correct responses. However, clustering and switching analysis can be used as an alternative method. Clustering involves the production of words within the same semantic subcategory, whereas switching involves shifting from one subcategory (cluster) to another^(3,18)^. While clustering relies on the semantic knowledge available in verbal working memory, switching relies on the processes that involve attention and executive functions^(4,18)^. There is evidence that people with impaired switching abilities have frontal lobe damage, whereas those with impaired clustering abilities have temporal lobe damage^(4,18)^.

In people with AD, clustering and switching analysis in verb fluency can be of particular relevance to understanding the cognitive nature of the verb retrieval deficits observed in this population. This knowledge, in turn, can provide essential information for a better understanding of the neurobiology of human language and, from a clinical perspective, for the development of an appropriate treatment plan in neuropsychological rehabilitation of people with AD. These alternative methods of verb fluency analysis, if used in combination with other assessment resources, may also be investigated for their utility as a tool for detection and diagnosis of AD in future studies. Therefore, the current study aimed to investigate verb fluency performance in individuals with AD compared with healthy older adults by analyzing total correct responses, number of clusters, average cluster size, and number of switches.

## METHODS

### Study design

This is a case-control study.

### Participants

Participants in this study were selected from the database of a larger project that included individuals with AD (AD group - ADG) and healthy older adults (control group - CG). Participants in the ADG were recruited from the neurodementia outpatient clinic of Hospital de Clínicas de Porto Alegre (HCPA), southern Brazil. The CG consisted of community-dwelling individuals participating in local social groups, matched with ADG participants for age, sex, and education. All participants, in both groups, were older than 65 years and native speakers of Brazilian Portuguese.

The ADG consisted of older adults with a neurological diagnosis of probable AD according to the Diagnostic and Statistical Manual of Mental Disorders (DSM-IV) and National Institute of Neurological and Communicative Disorders and Stroke - Alzheimer’s Disease and Related Disorders Association (NINCDS-ADRDA) criteria^(19)^ who were at the mild-to-moderate stage of AD. Neurological diagnosis included physical examination, laboratory testing, neuroimaging studies, and neuropsychological assessment. Dementia severity was determined using global Clinical Dementia Rating (CDR) scores (mild = CDR 1; moderate = CDR 2)^(20)^.

Controls underwent a brief interview for assessing their health conditions and functional independence. The Mini-Mental State Examination (MMSE) was also administered^(21)^. Only individuals with scores above the MMSE cutoff, without a history of neurological or psychiatric disorders, without a history of alcohol, substance, or benzodiazepine abuse, and without uncorrected visual or hearing impairment were included in the CG.

From the initial sample of 102 participants classified as having AD (ADG, n=40) or healthy controls (CG, n=62), those with missing data or who were unable to perform the verb fluency task due to difficulty understanding the task instructions were excluded from the current study. Therefore, the final sample analyzed in this study consisted of 68 participants, 29 in the ADG and 39 in the CG.

The study was approved by HCPA Research Ethics Committee (registration number 11-0178). Written informed consent was obtained from each study participant.

### Instrument and procedures

The verb fluency task was administered and recorded in a single session, after verifying all inclusion and exclusion criteria. Instructions for the verb fluency task were adapted from Piatt et al.^(8)^ to Brazilian Portuguese by Beber and Chaves^(7:32)^: “*I’ll set one minute, and during this time, I’d like you to tell me as many words as possible that mean things that people do - for example, eat and walk. Do you understand what I mean?*”. If the answer was yes: “*Can you please give me an example?*”. If the response was acceptable, the examiner stated: “*Now, let’s get started. Please tell me other words that mean things that people do, besides eat and walk.*”. If the response was unacceptable, the examiner repeated the instructions and provided another example. For individuals with a higher level of education, the words ‘verb’ or ‘action’ can be used to explain the task. During the task, the examiner made use of a recording protocol, timer, and recorder.

To obtain the total score for verbs correctly generated and for clustering and switching, the following procedures were performed: 1) development of a database of words retrieved by the participants; 2) operationalization of verb categories; 3) standardization of the selection of measures between raters; 4) categorization of words and selection of the variables by independent raters; 5) analysis of inter-rater reliability; 6) new categorization of discordant items; and 7) final version of the categorization of words retrieved.

Step 1: Development of a database of retrieved words

For the present study, verbs produced in one minute by older adults were used in the verb fluency task. The verbs produced by each participant were entered into a Microsoft Excel spreadsheet.

Step 2: Operationalization of verb categories

To define which verb categories would compose the clusters, three different raters independently categorized the verbs produced by 20 participants (10 in the ADG and 10 in the CG). Responses were divided into two major categories: observable and unobservable actions. This was based on the procedure for categorization of verbs used in a previous study of adults with schizophrenia vs healthy adults^(22)^, which considered only two superordinate categories: action verbs, which included verbs expressing concrete, observable actions; and mental state verbs, which included verbs whose meaning relates to understanding, discovering, planning, or deciding and verbs of perception, cognition, and emotion (unobservable verbs). This categorization was also used by Paek and Murray^(23)^ in a sample of individuals with AD and healthy older adults.

In the present study, additionally, the verbs within each major category were divided into subcategories according to the semantic clustering strategies used by the participants and observed by the raters:

*Observable actions:* 1) actions related to body parts (those associated with the movement of the legs, hands, and mouth and those associated with communication); 2) routine actions (home activities, self-care, and sports, leisure, and social interaction activities); and 3) actions with objects (possession, production).

*Unobservable actions:* 1) psychological actions (senses and feelings, intellectual activities, religiosity and values); and 2) verbs of existence and auxiliaries.

The raters then met to discuss these categories and to determine which categories would be used and which words would be accepted in each category.

Step 3: Standardization of the selection of measures between raters

Two different raters independently selected word clusters from the verbs retrieved by 10 participants (5 in the ADG and 5 in the CG) according to the categories established in the previous step. For each participant, the raters scored the total number of clusters, average cluster size, and number of switches. Subsequently, the raters discussed these measures in order to standardize the selection of categories and participants’ scores. A table containing examples of verbs belonging to each category was developed (Chart 1).

**Chart 1:** Verb categories and their respective examples

**Category**	**Subcategory**	**Cluster**	**Examples**
Observable actions	Actions related to body parts	Movement of the legs/feet	run, jog, dive, sink, depart, walk, climb, drop, escape, return, travel, leave, stroll, go, run, jump, fall, tumble, enter, fly, reach, crawl, leap, swim, drive, ride (a bike), come, slip away, sit, get up, jump, tap dance
		Movement of the hands	lift, open, close, pour, grab, draw, fix, fetch, bring, take out, put in, release, fasten, take, sew, cook, paint, keep, hide, cover up, carry, put on
		Communication	search, send (an email), receive, log in, dial, phone, communicate, speak, listen
		Movement of the mouth	eat, chew, have lunch, swallow, talk, sing, chat, rant, scream, drink, breathe, cough, smile, count, whistle, call, curse, ingest, digest, laugh, dine
	Routine actions	Home activities	sweep, wash, dry, cook, sew, wash (the dishes), prepare (food), soil, clean, iron, make (coffee), clean (the house), sweep (the yard), wash (clothes), take care (of the dogs), give (bath), take care of, tidy up
		Self-care	bathe, paint (nails), put on (makeup), eat, sleep, wake up, dress, shave, dream, drink (water), brush (teeth), get up, comb, walk, jog, visit (a doctor), take (medicine)
		Sports, leisure, and social interaction activities	stroll, go (to church), sing, dance, play, summer, hunt, fish, walk, fight, run, do (gymnastics), move, exercise, dance, sing, travel, dive, swim, jog, play, ride (a bike), play, kick, catch, stroll, visit, listen, date, dine, see (my grandchildren), go (to the movies)
	Actions with objects	Possession	sell, buy, shop (for groceries), go shopping, receive (money), possess, have, use, lose, acquire
		Production	sew, paint, work, increase, decrease, fabricate, provide, slice, close, pierce, moisten, rotate, paint, clean, air, make, complete, pick up, spank, form, integrate, sew, crochet, embroider, sweep, open, polish, blow, bake, decorate, mix, chop, knead, nibble, grate
Unobservable actions	Psychological actions	Feelings	smile, cry, love, feel, hug, welcome, suffer, date, fall in love, please, forgive, desire, be (happy), hurt, harm
		Senses	notice, observe, look at, see, watch, speak, hear, attend, despise
		Intellectual activities	watch (TV), read, study, believe, acknowledge, know, listen, recognize, paint, draw, guess, report, rewrite, write, work, translate, draft
		Religiosity and values	sing, pray, help, do (good to humanity), assist (friends), do (volunteer work), support, love
	Verbs of existence and auxiliaries		there be, continue, be, stay, stop, dwell, go

Step 4: Categorization of words retrieved and selection of clusters and switches by independent raters

After determining the measures, the two raters trained in the previous step independently analyzed the verbs produced by each participant, blinded to which group individual participants had been allocated. The raters determined the number of clusters, average cluster size, number of switches, and total number of verbs retrieved by each participant. The selection process for each variable is described below.

*Total number of correct words retrieved:* total number of words retrieved, excluding repetitions and errors^(7)^. Repetitions were defined as verbs mentioned more than once. Errors were defined as words not morphologically classified as verbs (e.g., glass, beautiful). In addition, the verbs ‘eat’ and ‘walk’ were also considered errors when participants retrieved them as one of the first two successively generated words, because both were used as example verbs in the task instructions. For example, if a participant retrieved “eat, walk, travel, jog, leave, sleep, travel, leave,” a total of four words retrieved would be correct (travel, jog, leave, and sleep).

*Number of clusters:* total number of clusters for each participant. The items generated by each participant were considered to form a cluster when at least two successively generated words belonged to the same category. Single words were not considered clusters. For example, the sequence “walk, jog, comb, climb, drop, escape, sweep, wash, dry, dress, comb” shows a total of four clusters (walk and jog; climb, drop, and escape; sweep, wash, and dry; dress and comb) and one single word (comb). Different from the total number of words retrieved, errors and repetitions were included in the calculation of number of clusters, as these data provide important information about the strategies and cognitive processes used by the participants^(18)^.

*Average cluster size:* cluster size was counted beginning with the second word produced in each cluster (e.g., “walk and jog” is size one; “climb, drop, and escape” is size two; “sweep, wash, and dry” is size two; and “dress and comb” is size one). Average cluster size was calculated by summing the sizes of each cluster produced by each participant and dividing this value by the total number of clusters for each participant. Therefore, in the example above, the average cluster size for the participant would be 1.5 (obtained by dividing six by four). Errors and repetitions were also included in the calculation of average cluster size.

*Number of switches:* switches were calculated as the number of transitions between clusters, including single words. Using again the example above, the sequence “walk, jog, comb, climb, drop, escape, sweep, wash, dry, dress, comb” shows a total of four switches (transitions between “jog and comb,” “comb and climb,” “escape and sweep,” and “dry and dress”). Errors and repetitions were also included in this measure, that is, the total production of each participant was considered.

Step 5: Analysis of inter-rater reliability

Inter-rater reliability for each variable scored by the two independent raters (total number of correct words retrieved, number of clusters, average cluster size, and number of switches) was determined by the intraclass correlation coefficient (ICC)^(24)^, using the R irr package^(24,25)^. ICC values ≥ 0.75 were considered excellent correlations^(24)^. The following ICC values were obtained: 0.982 for number of clusters, 0.958 for average cluster size, and 0.992 for number of switches.

Step 6: New categorization of discordant items

After inter-rater reliability analysis, discordant items between the two raters were reanalyzed one by one by two newly trained raters, who independently categorized these items. The scores of the two new raters were used to reach a decision on the final score for these items.

Step 7: Final version of the categorization of variables

Considering all the steps, all the final scores for each participant were defined and tabulated for analysis in the four study variables (total number of correct words retrieved, number of clusters, average cluster size, and number of switches).

### Data analysis

Categorical variables were expressed as absolute and relative frequencies, and continuous variables were expressed as mean (SD). The descriptive characteristics of the groups were compared using Student’s t test and the chi-square test. Study outcomes (total number of correct words retrieved, number of clusters, average cluster size, and number of switches) were compared between groups by analysis of covariance (ANCOVA) using age and education as covariates. Data were analyzed using SPSS, version 25. The ICC was used to determine inter-rater reliability, using the R irr package^(25)^. The level of significance was set at 5% for all analyses.

## RESULTS

Table 1 shows the descriptive data of the sample and significant differences between the CG and ADG in terms of age and education. On average, ADG participants were older than controls, whereas controls had a higher level of education than ADG participants.

**Table 1:** Sample description per group

**Variable**	**CG (n=39)**	**ADG (n=29)**	**p (CG x ADG)**
Age (years), mean (SD)	71.5 (6.3)	78.6 (6.7)	<0.01*
Education (years), mean (SD)	7.1 (3.4)	3.8 (3.1)	<0.01*
Sex (F), n (%)	34 (87.2)	20 (69)	0.07

* Significant at p ≤ 0.05, analysis of covariance

**Caption:** ADG = Alzheimer’s Disease Group; CG = Control Group; F = Female; SD = Standard Deviation

Participants in the CG and ADG were then compared for the study outcomes (total number of correct words retrieved, number of clusters, average cluster size, and number of switches), controlling for differences in age and education, which were included as covariates in the statistical analysis. Table 2 shows the results of these comparisons, where the mean number of switches and total number of verbs retrieved were significantly lower in the ADG.

**Table 2:** Verb fluency performance per group

**Variable**	**CG (n=39)**	**ADG (n=29)**	**p**
Number of clusters, estimated mean (SD)	2.4 (0.2)	2.1 (0.3)	0.40
Cluster size, estimated mean (SD)	1.6 (0.1)	1.5 (0.2)	0.60
Number of switches, estimated mean (SD)	5.5 (0.5)	3.9 (0.6)	0.05*
Total number of verbs produced, estimated mean (SD)	9.6 (0.6)	7.0 (0.7)	0.01*

* Significant at p ≤ 0.05, analysis of covariance

**Caption:** ADG = Alzheimer’s Disease Group; CG = Control Group; SD = Standard Deviation

Additionally, the study outcomes were compared only within the ADG by comparing participants with mild vs moderate AD. There was no statistically significant difference in any of the variables analyzed (Table 3).

**Table 3:** Comparison of study outcomes between individuals with mild and moderate AD

**Variable**	**Mild AD (n=18)**	**Moderate AD (n=11)**	**p**
Number of clusters, estimated mean (SD)	2.1 (0.3)	1.3 (0.4)	0.15
Cluster size, estimated mean (SD)	1.7 (0.2)	1.3 (0.3)	0.28
Number of switches, estimated mean (SD)	3.5 (0.6)	2.4 (0.7)	0.24
Total number of verbs produced, estimated mean (SD)	7.0 (0.7)	5.0 (0.9)	0.09

**Caption:** AD = Alzheimer’s Disease; SD = Standard Deviation

## DISCUSSION

This study aimed to investigate verb fluency performance in individuals with AD compared with cognitively healthy older adults. To this end, we used a thorough methodological approach in categorizing verbs according to semantic criteria in order to obtain the study variables: total number of correct words retrieved, number of clusters, average cluster size, and number of switches. The procedure for categorization of verbs was based on the classification proposed by Smirnova et al.^(22)^ which was also used in a previous study of individuals with AD and healthy older adults^(23)^. However, our procedure differed slightly in that the verbs were divided into subcategories within the two major categories proposed by these authors. We made this decision after observing that the verbs generated by our participants and the semantic strategies employed by them required a more refined classification of verb categories in order not to miss the wealth of information provided by our sample.

As for the comparison of the outcomes of interest between the CG and ADG, our data suggest that there is a quantitative difference in the production of verbs between the groups, with a lower word production in individuals with AD than in controls, as described in previous studies^(11,13,14)^. Verbs are considered the grammatical class that most requires a complex semantic organization for retrieval and, therefore, would be the most sensitive to deficits.

In the present study, the ADG and CG differed in the number of switches, but not in the number and size of clusters. This is consistent with the findings from previous studies comparing semantic fluency performance between healthy older adults and individuals with AD in terms of clustering and switching strategies^(10,26,27)^. A reduced number of switches suggests that individuals with AD have limited organizational strategies for verb retrieval and, possibly, retrieve a reduced number of verbs as a consequence. Therefore, this specific impairment in the verb fluency task would occur as a result of executive function deficits and not necessarily due to difficulties in lexical-semantic access to this class of words (or, at least, the executive difficulties in this task would exceed those of the lexical-semantic system).

The poorer performance of older adults with AD (vs without AD) in verb switching may result from working memory deficits. A previous study investigated the relationship between verbal fluency performance and working memory performance in older people with and without cognitive decline by means of clustering and switching analysis^(4)^. The decrease in switching ability was associated with the decline in working memory^(4)^. In another study of adults over 50 years of age with neurodegenerative disease, switching strategies in verbal fluency also proved to be sensitive to pathological changes in executive abilities^(13)^.

Another hypothesis is that verb fluency deficits would be related to language deficits. A study compared the performance of healthy older adults, older adults with mild AD, and older adults with moderate AD in verb fluency and verb naming tasks and concluded that patients with AD were equally impaired in verb fluency and verb naming^(12)^. However, when compared in terms of AD severity, those with more advanced disease performed more poorly in the verb naming task, but scores did not differ in the verb fluency task^(12)^, as also observed in the present study. The authors suggested that, in individuals with AD, deficits in verb processing have a predominantly semantic nature, which does not exclude the influence of impairments in other cognitive domains^(12)^. As the performance in verb processing also depends on the task used for verb retrieval, the results of the present study indicate that, in the verb fluency task, the ADG’s poorer performance was mostly driven by executive dysfunction, demonstrating that this task is more sensitive to executive impairment than to disruption in semantic verb processing.

Furthermore, although they were not the focus of our study, some qualitative differences were observed in verb production between the study participants. Overall, participants with AD used complementary words to describe the verbs, which appears to be directly related to the quality of the verb, retrieving verbs that can be used more generally and in less specific contexts (e.g., ‘prepare food’ and ‘go shopping’). This may suggest difficulty retrieving less frequent verbs in Portuguese. Also, participants may have had difficulty understanding the task itself. This may have occurred at the time they were given the instructions or in the process of holding the instructions in mind, which suggests a decline in working memory capacity. However, the qualitative data obtained in this study are insufficient to further interrogate this topic, thus being reported as secondary results of the research. Future research along these lines is warranted to further explore this phenomenon.

The assessment of clustering included repetitions, which differs from the traditional assessment of data obtained in fluency tasks that considers only the total number of non-repeated words. This decision was based on the assumption that clustering analysis may help identify word retrieval strategies used by the participants. The present data suggest that individuals with AD use fewer semantic strategies than healthy controls, retrieving verbs that are apparently unconnected. Despite the clear correlation between the number of words retrieved and number of clusters, the first measure loses its explanatory power at this level of analysis.

Finally, our study provides evidence that verb fluency performance, in terms of total number of verbs retrieved and switching ability, is impaired in individuals with AD. Specifically in this task, verb fluency deficits may be predominantly executive in nature, highlighting the participants’ difficulty using cognitive strategies to retrieve verbs. These findings can be useful in different ways. First, both the verb fluency task itself and the switching ability in this task may be markers of conversion to AD, but not necessarily of disease progression (since there was no significant difference in the outcomes between individuals with mild and moderate AD). Second, this cognitive feature may be relevant to treatment planning in neuropsychological rehabilitation. For example, in interventions aimed at reducing anomies, training in the use of different strategies to retrieve verbs/actions can help patients access these words more easily. Third, our findings suggest that temporal lobe structures (affected in the early stages of AD) and their connections are also important in the search for cognitive strategies to retrieve verbs.

Additional investigation is needed to further explore this topic, including studies with larger sample sizes that also investigate the different presentations of AD (e.g., amnestic and dysexecutive profiles), as this was not possible in the present study. The methods used here might be applied to populations with other neurological diseases or even to healthy individuals of different ages and levels of education in order to understand more clearly the neural processing of verbs and the utility of the verb fluency task.

## CONCLUSION

The current study showed that older adults with AD have impairments in both total number of correct verbs retrieved during the verb fluency task and switching ability compared with healthy older adults. The results indicate that verb fluency is more sensitive to cognitive deficits resulting from the executive dysfunction than from the semantic disruption observed in people with AD.

In clinical settings, the verb fluency task may be used as part of cognitive assessment in individuals with AD, also assisting in the diagnosis, monitoring and decision-making for neuropsychological rehabilitation through the switching and clustering measures. In research settings, the methods used in the current study may be further explored in other neurological diseases in order to understand their cognitive profiles.
