# Supplementary material for: Clustering and switching analysis of verb fluency in individuals with Alzheimer’s disease
Source: Codas. 2023 Apr 17;35(2):e20210179. doi: 10.1590/2317-1782/20232021179en (PMC10145992; doi:10.1590/2317-1782/20232021179en)
Supplement: Translation

# Translation: Análise de clustering e switching da fluência de verbos em indivíduos com doença de Alzheimer

**DOI:** 10.1590/2317-1782/20232021179pt

**Authors:** Bárbara Costa Beber, Franceia Veiga Liedtke, Felipe Schroeder de Oliveira, Lucas Müller-Silveira, Emily Viega Alves, Márcia Lorena Fagundes Chaves, Jerusa Fumagalli de Salles

**Affiliations:** 1 Departamento de Fonoaudiologia, Universidade Federal de Ciências da Saúde de Porto Alegre - UFCSPA - Porto Alegre (RS), Brasil.; 2 Programa de Pós-graduação em Ciências da Reabilitação, Universidade Federal de Ciências da Saúde de Porto Alegre - UFCSPA - Porto Alegre (RS), Brasil.; 3 Programa de Pós-graduação em Psicologia, Universidade Federal do Rio Grande do Sul - UFRGS - Porto Alegre (RS), Brasil.; 4 Curso de Psicologia, Universidade Franciscana - UFN - Santa Maria (RS), Brasil.; 5 Curso de Fonoaudiologia, Universidade Federal de Ciências da Saúde de Porto Alegre - UFCSPA - Porto Alegre (RS), Brasil.; 6 Departamento de Medicina Interna, Programa de Pós-graduação em Medicina - Ciências Médicas, Universidade Federal do Rio Grande do Sul - UFRGS - Porto Alegre (RS), Brasil.; 7 Departamento de Psicologia do Desenvolvimento e da Personalidade, Universidade Federal do Rio Grande do Sul - UFRGS - Porto Alegre (RS), Brasil.

**Keywords:** Doença de Alzheimer, Cognição, Linguagem, Neuropsicologia, Demência

## Abstract

**Objetivo:**

Investigar o desempenho na tarefa de fluência de verbos de pessoas com doença de Alzheimer em comparação à idosos saudáveis, a partir da análise do número total de palavras corretas evocadas, do número de *clusters,* do tamanho médio dos *clusters* e do número de *switches*.

**Método:**

Este é um estudo de caso-controle no qual participaram 39 idosos saudáveis e 29 participantes com diagnóstico de doença de Alzheimer. O desempenho da fluência de verbos dos participantes foi analisado quanto ao total de verbos gerados corretamente, número de *clusters*, tamanho médio dos *clusters* e número de *switches*. Para a obtenção dos desfechos do estudo foi realizado um processo prévio de definição das categorias de verbos que constituiriam clusters. A classificação dos verbos foi adaptada para a este estudo, incluindo a análise de juízes com investigação do índice de concordância.

**Resultados:**

Dentre as variáveis de interesse comparadas entre os grupos estudados, houve um desempenho estatisticamente inferior no grupo de pessoas com doença de Alzheimer quanto ao número de *switches* e total de verbos gerados corretamente. Nas demais medidas, os grupos foram semelhantes.

**Conclusão:**

Neste estudo pessoas com doença de Alzheimer apresentaram déficit no desempenho da tarefa de fluência verbal de verbos, caracterizado por uma redução do número total de verbos gerados e uma menor variabilidade de categorias de verbos. Os achados sugerem que, na doença de Alzheimer, a fluência de verbos é mais sensível aos déficits cognitivos decorrentes de disfunção executiva do que aos déficits semânticos.

## INTRODUÇÃO

A fluência verbal é uma tarefa de avaliação neuropsicológica na qual o indivíduo deve evocar, geralmente durante um minuto, o maior número de palavras de acordo com uma dada característica ou critério. Os tipos de fluência verbal, de acordo com o critério de evocação das palavras são: fluência verbal semântica (geração de palavras de uma categoria semântica específica, como animais ou frutas, por exemplo), fluência verbal fonêmica ou ortográfica (geração de palavras que começam como uma determinada letra, sendo as letras “f”, “a” e “s” as mais utilizadas), fluência de verbos ou de ações (geração de verbos ou palavras que representam “coisas que as pessoas podem fazer”), e a fluência verbal livre^(1-3)^. De modo geral, as tarefas de fluência verbal exigem a utilização de habilidades cognitivas de linguagem, memória semântica, funções executivas e memória de trabalho^(4)^. No entanto, acredita-se que cada tipo de tarefa pode demandar mais de determinado processo cognitivo e recrutar determinadas áreas cerebrais de acordo com o critério utilizado para a geração das palavras^(5,6)^. Pode-se afirmar também que altos níveis de desempenho cognitivo geral e educacional estão associados com um melhor desempenho nas tarefas de fluência verbal^(5,6)^.

A fluência verbal de verbos é mais complexa do que as tarefas de fluência verbal que envolvem a geração de substantivos, pois os verbos possuem maior inflexão e maior relação sintática com outras palavras^(7)^. Além disso, quando evocados, os substantivos e verbos ativam áreas distintas no cérebro. A evocação de substantivos comuns e nomes próprios são mediadas predominantemente e respectivamente pelas regiões temporal posterior e anterior do cérebro^(8,9)^. Já a evocação de verbos ocorre mediada, principalmente, pelas regiões frontais do cérebro, o que a torna um potencial indicador na avaliação das habilidades ligadas às funções executivas^(8,9)^. A fluência de verbos tem sido sugerida pela literatura como um marcador de comprometimento frontoestriatal pela sua sensibilidade à integridade destas redes do cérebro e como uma nova medida de funcionamento executivo e linguístico^(7,9)^.

As tarefas de fluência verbal, em geral, são muito utilizadas na avaliação cognitiva de sujeitos com doenças neurodegenerativas. Na doença de Alzheimer (DA), já foram descritos déficits na realização de tarefas de fluência verbal, especialmente na semântica, que costuma estar mais afetada que a ortográfica^(10-12)^. Essas características estão relacionadas a um predomínio do comprometimento de regiões do lobo temporal esquerdo, especificamente ligadas ao processamento da memória semântica^(11,13,14)^. Já os estudos que investigaram a fluência de verbos na DA encontraram uma pior performance em relação aos seus pares saudáveis^(12,15)^, em relação a sujeitos com comprometimento cognitivo leve^(16)^ e esta tarefa também foi um indicativo da conversão de indivíduos cognitivamente saudáveis em comprometimento cognitivo leve^(17)^. Ainda, é preciso elucidar melhor qual a natureza cognitiva dos déficits na fluência de verbos na DA, se ocorre no nível semântico ou executivo, ou em ambos. Para isso, métodos que analisam com mais profundidade as características dos verbos evocados e a forma de evocação (agrupamentos ou *clusters*, e mudança de categorias), para além do número total de verbos gerados apenas, podem ser de grande utilidade.

A forma mais tradicional para se analisar a performance nas tarefas de fluência verbal, incluindo a fluência de verbos, é o escore total de palavras emitidas de forma correta. Porém, outra metodologia possível é a análise de *clustering* (agrupamento) e *switching* (alternância). Os *clusters* são agrupamentos de palavras com a mesma subcategoria semântica. Já a alternância ou *switching* ocorre quando um *cluster* termina e se inicia outro *cluster* ou uma palavra isolada^(3,18)^. O processo de *clustering* depende do conhecimento semântico disponível na memória de trabalho, já o *switching* depende dos processos que envolvem a atenção e funções executivas^(4,18)^. Existem evidências de que pessoas com problemas em realizar o *switching* tenham maior comprometimento de áreas frontais do cérebro, enquanto que as com problemas para realizar o *clustering* tenham maior comprometimento de áreas temporais^(4,18)^.

A análise de *clustering* e *switching* na fluência de verbos de pessoas com DA pode ser de grande relevância para compreender a natureza cognitiva do déficit na evocação de verbos que ocorre nessa população. Por consequência, compreender a natureza cognitiva desta dificuldade fornece subsídios para uma melhor compreensão da neurobiologia da linguagem humana e, do ponto de vista clínico, para um adequado planejamento terapêutico da reabilitação neuropsicológica das pessoas com DA. Ainda, outros métodos de análise da fluência de verbos podem ser investigados pensando em seu uso como ferramenta de detecção e diagnóstico da DA, obviamente em combinação com outros recursos avaliativos. Sendo assim, este estudo teve como objetivo investigar o desempenho na tarefa de fluência de verbos de pessoas com DA em comparação à idosos saudáveis, a partir da análise do número total de palavras corretas evocadas, do número de *clusters*, do tamanho médio dos *clusters* e do número de *switches*.

## MÉTODO

### Desenho do estudo

Este estudo se caracteriza como um estudo de caso-controle.

### Participantes

Os participantes foram selecionados a partir da análise de um banco de dados de um projeto maior que possuía participantes classificados em grupo com DA (GDA) e grupo de idosos saudáveis (grupo controle - GC).

Os participantes do GDA foram recrutados do ambulatório de neurodemências do Hospital de Clínicas de Porto Alegre (HCPA), RS. Já o GC foi composto por indivíduos recrutados de grupos sociais da comunidade local, procurando manter um perfil semelhante ao GDA quanto a idade, sexo e escolaridade. Para ambos os grupos, só foram incluídos participantes falantes do português brasileiro com idade maior que 65 anos.

No GDA foram incluídos apenas adultos idosos que receberam o diagnóstico neurológico de DA provável a partir dos critérios diagnósticos do *Diagnostic and Statistical Manual of Mental Disorders (DSM-IV) e National Institute of Neurological and Communicative Disorders and Stroke - Alzheimer's Disease and Related Disorders Association* (NINCDS-ADRDA)^(19)^ e que estavam em estágio leve e/ou moderado da DA. Para o diagnóstico neurológico, foram realizados além do exame físico, exames laboratoriais e de neuroimagem, e uma avaliação neuropsicológica. A gravidade da demência foi avaliada por meio de pontuações globais do *Clinical Dementia Rating* (CDR) (leve = CDR 1; moderado = CDR 2)^(20)^.

Para o GC, foi realizada uma breve entrevista que objetivava a verificação das condições de saúde e independência funcional dos participantes. Além disso, foi aplicado o Mini-Exame do Estado Mental (MEEM)^(21)^. Foram incluídos no GC apenas indivíduos com pontuação acima do ponto de corte no MEEM, sem histórico de problemas neurológicos ou transtornos psiquiátricos ou abuso de álcool, drogas ou benzodiazepínicos; e sem deficiência visual ou auditiva não corrigida.

Da amostra inicial de 102 participantes já classificados em GDA (n= 40) e GC (n= 62), foram excluídos, especificamente para este estudo, os participantes que possuíam dados faltantes e que não conseguiram executar a tarefa da fluência de verbos por dificuldade na compreensão da ordem. Deste modo, a amostra final deste artigo foi composta por 68 indivíduos (GDA=29; GC=39).

Este estudo foi aprovado pelo Comitê de Ética em Pesquisa do HCPA (número de registro 11-0178) e todos os participantes assentiram em participar do estudo através da assinatura de um Termo de Consentimento Livre e Esclarecido.

### Instrumento e procedimentos

A tarefa de fluência de verbos foi coletada e gravada em uma única sessão, após a verificação de todos os critérios de inclusão e exclusão. Nessa tarefa, os participantes ouviram a seguinte instrução, adaptada para o português brasileiro de Piatt et al.^(8)^ por Beber e Chaves^(7:32)^: “*Irei marcar um minuto, e durante este tempo você terá que dizer o máximo de palavras que significam coisas que podemos fazer, como por exemplo, comer e andar. Você entendeu?*”. Se a resposta for sim: “*Dê-me um exemplo, então.*”. Se o exemplo estiver correto: “*Agora, iremos começar. Diga-me outras palavras que representam coisas que podemos fazer, além de comer e andar*.”. Se o exemplo dado estiver incorreto, dê a explicação novamente com mais um exemplo. Para pessoas com maior escolaridade podem ser utilizados os termos “verbo” ou “ação” para explicar a tarefa. Durante a tarefa, o examinador fez uso de um protocolo de registro, cronômetro e gravador.

Para a extração do escore total de verbos gerados corretamente e das variáveis de *clustering* e *switching* foram realizadas os seguintes procedimentos: 1) elaboração do banco de dados com as palavras evocadas pelos participantes; 2) operacionalização das categorias de verbos; 3) uniformização da extração de medidas entre avaliadores; 4) categorização das palavras e extração das variáveis pelos juízes de forma independente; 5) análise de fidedignidade da análise pela concordância entre juízes; 6) nova categorização dos itens discordantes e 7) versão final da categorização das palavras evocadas.

Etapa 1: Elaboração do banco de palavras evocadas

Para esse estudo, foram utilizados verbos evocados durante um minuto pelos adultos idosos na tarefa de fluência verbal de verbos. Os verbos evocados por cada um dos participantes foram transcritos para uma planilha do Microsoft Excel.

Etapa 2: Operacionalização das categorias de verbos

Para a definição de quais categorias de verbos iriam compor os *clusters*, três diferentes juízes categorizaram de forma independente os verbos gerados por 20 participantes (10 GDA e 10 GC). Organizou-se as palavras em duas grandes categorias: ações observáveis e não observáveis. Esse agrupamento baseou-se na categorização usada em um estudo prévio^(22)^ que organizou verbos evocados por uma amostra de adultos com esquizofrenia e adultos saudáveis. Os autores consideraram apenas duas grandes categorias: a primeira que incluía verbos que exprimem ações concretas, observáveis e a segunda que contemplava verbos cujo significado estão relacionados à compreensão, descobertas, planejamentos, decisões, percepção, cognição e emoção (verbos não observáveis). Essa forma de categorizar já foi utilizada também por Paeka e Murray^(23)^ com uma amostra de idosos com DA e saudáveis^(24)^.

Adicionalmente, os verbos de cada grande categoria foram distribuídos em subcategorias, de acordo com as estratégias de agrupamento semântico utilizadas pelos participantes e observadas pelos juízes:

*Ações observáveis:* 1) ações de corpo (deslocamento e movimento de pernas; deslocamento e movimento de mãos; movimentos de comunicação; movimentos da boca), 2) ações de rotina (cuidados da casa; autocuidado; esporte, lazer e convívio social), 3) ações com objetos (posse; produção).

*Ações não observáveis:* 1) ações psicológicas (sentidos, sentimentos; intelectual; religiosidade e virtudes) e 2) verbos de existência e auxiliares.

A seguir, as categorias criadas foram discutidas pelos juízes, que definiram quais categorias seriam utilizadas e quais palavras seriam aceitas em cada uma delas.

Etapa 3: uniformização da extração de medidas entre avaliadores

Nessa etapa, dois diferentes avaliadores extraíram, de forma independente, dentre os verbos evocados por 10 participantes (5 GA e 5 GC), grupos de palavras que formaram clusters, conforme as categorias estabelecidas na etapa anterior. Após, pontuaram, para cada um dos participantes a quantidade total de *clusters*, o tamanho médio dos *clusters* e a quantidade de *switches*. As medidas então foram discutidas entre os avaliadores, a fim de uniformizar a extração de categorias e pontuação dos participantes. Nessa fase, foi organizada uma tabela com exemplos de verbos pertencentes a cada categoria (Quadro 1).

**Quadro 1:** Categorias de verbos e seus respectivos exemplos

**Categoria**	**Subcategoria**	**Clusters**	**Exemplos**
Ações Observáveis	Ações de corpo	Deslocamento e movimentos de pés pernas	correr, caminhar, mergulhar, afundar, afastar, andar, subir, descer, fugir, voltar, viajar, sair, passear, ir, correr, saltar, cair, tombar, entrar, voar, chegar, engatinhar, pular, nadar, dirigir, pedalar, vir, escapulir, sentar, levantar, pular, sapatear
		Deslocamento e movimentos de mãos	levantar, abrir, fechar, servir, agarrar, desenhar, corrigir, buscar, trazer, tirar, colocar, soltar, prender, levar, costurar, cozinhar, pintar, guardar, esconder, encobrir, transportar, colocar
		Movimentos de comunicação	pesquisar, passar (e-mail), receber, entrar (no facebook), discar, telefonar, comunicar, falar, ouvir
		Movimentos da boca	comer, mastigar, almoçar, engolir, falar, cantar, soltar, esbravejar, gritar, beber, respirar, tossir, sorrir, contar, assoviar, chamar, xingar, deglutir, digerir, rir, jantar
	Ações de rotina	Cuidados da casa	varrer, lavar, secar, cozinhar, costurar, lavar (a louça), fazer (comida), sujar, limpar, passar, fazer (café), lavar (a casa), varrer (o pátio), lavar (roupa), cuidar (dos cachorros), dar (banho), cuidar, arrumar.
		Autocuidado	tomar (banho), pintar (as unhas), pintar (o rosto), comer, dormir, acordar, vestir, fazer (a barba), sonhar, tomar (água), escovar (os dentes), levantar, pentear, caminhar, andar, consultar, tomar (remédio)
		Esporte, lazer e convívio social	passear, ir (à missa), cantar, dançar, jogar, veranear, caçar, pescar, andar, lutar, correr, fazer (ginástica), movimentar, fazer (exercícios), dançar, cantar, viajar, mergulhar, nadar, caminhar, brincar, pedalar, jogar, chutar, pegar, Passear, visitar, ouvir, namorar, jantar, ver (meus netos), ir (ao cinema).
	Ações com objetos	Posse	vender, comprar, ir (no armazém), fazer (compras), receber (dinheiro), possuir, ter, usar, perder, adquirir
		Produção	costurar, pintar, trabalhar, aumentar, diminuir, fabricar, fornecer, fatiar, fechar, furar, umedecer, girar, pintar, limpar, arejar, fazer, completar, apanhar, surrar, formar, integrar, costurar, fazer (crochê), bordar, varrer, abrir, polir, ventar, cozer, embelezar, misturar, picar, amassar, roer, ralar
Ações não observáveis	Ações psicológicas	Sentimentos	sorrir, chorar, amar, sentir, abraçar, acolher, sofrer, namorar, apaixonar, agradar, perdoar, desejar, ser (feliz), ferir, machucar
		Órgãos dos sentidos	constatar, observar, olhar, ver, assistir, falar, ouvir, atender, desprezar.
		Atividades intelectuais	ver (TV), ler, estudar, crer, conhecer, saber, ouvir, reconhecer, pintar, desenhar, adivinhar, relatar, reescrever, redigir, trabalhar, traduzir, escrever
		Religiosidade e virtudes	cantar, orar, auxiliar, fazer (o bem à humanidade), ajudar (os amigos), fazer (trabalho voluntário), ajudar, amar
	Verbos de existência e auxiliares		haver, continuar, ser, estar, ficar, parar, morar, ir

Etapa 4: Categorização das palavras evocadas e extração das variáveis *clusters* e *switches* pelos juízes de forma independente

Após a aferição das medidas, os dois juízes avaliadores treinados na etapa anterior analisaram os verbos produzidos por cada um dos participantes de forma independente e cega quanto ao grupo em que o participante estava alocado. Os juízes contabilizaram o número de *clusters*, tamanho médio dos *clusters*, número de *switches*, e o número total de verbos evocados por cada participante. Segue abaixo como foram extraídas cada uma das variáveis utilizadas.

*Número total de palavras corretas evocadas:* quantidade total de palavras evocadas, excluindo-se repetições e erros^(7)^. As repetições são verbos mencionados mais de uma vez. Já os erros são as palavras não classificadas morfologicamente como verbos (ex: copo, bonito). Além disso, também são erros os verbos “comer” ou “andar” nas situações em que os participantes os evocaram entre os dois primeiros itens da sequência, em razão de ambos os verbos fazerem parte do *rapport* da tarefa. Assim, por exemplo, na evocação “comer, andar, viajar, caminhar, sair, dormir, viajar, sair” tem-se um total de quatro palavras corretas evocadas (viajar, caminhar, sair, dormir).

*Número de clusters:* quantidade total de clusters de cada participante. Considerou-se que os itens gerados por cada participante formam um *cluster* quando pelo menos duas palavras evocadas em sequência pertenciam à mesma categoria. Palavras isoladas não foram consideradas clusters. Considerando o seguinte exemplo, “andar, caminhar, pentear, subir, descer, fugir, varrer, lavar, secar, vestir, pentear”; conclui-se que há um total de 4 *clusters* (andar e caminhar; subir, descer e fugir; varrer, lavar e secar; vestir e pentear) e 1 palavra isolada (pentear). Diferente da quantidade total de palavras evocadas, para o número de *clusters* foram incluídos erros e repetições, em razão de esses dados fornecerem indícios importantes sobre as estratégias e processos cognitivos utilizados pelos participantes^(18)^.

*Média do tamanho dos clusters:* para calcular o tamanho de cada cluster, conta-se a quantidade total de palavras a partir da segunda palavra produzida (ex: “andar e caminhar” tem tamanho um; “subir, descer e fugir” tem tamanho dois; “varrer, lavar, secar” tem tamanho dois; e “vestir, pentear” tem tamanho um). Para a média do tamanho de *clusters*, somou-se os tamanhos de cada um dos *clusters* produzidos por cada participante e dividiu-se esse valor pelo número total de clusters de cada participante. Assim, no exemplo acima, a média do tamanho de clusters desse participante seria de 1,5 (obtido por intermédio da divisão entre seis e quatro). Da mesma forma que no total de *clusters*, os erros e as repetições foram considerados para formar a média do tamanho dos *clusters*.

*Número de switches:* para essa medida, analisa-se o número total de trocas entre *clusters* e/ou palavras isoladas. Tomando como exemplo novamente a sequência “andar, caminhar, pentear, subir, descer, fugir, varrer, lavar, secar, vestir, pentear”. Nesse caso, tem-se quatro *switches* (trocas de estratégia entre “caminhar e pentear”, “pentear e subir”, “fugir e varrer”, “secar e vestir”). Nessa medida, também foram considerados os erros e as repetições, ou seja, a produção total de cada participante.

Etapa 5: Análise de fidedignidade das análises pela concordância entre juízes

A fim de verificar a fidedignidade das análises feitas para cada variável pontuada pelos dois juízes independentes (número total de palavras corretas evocadas, número de *clusters*, média do tamanho dos *clusters* e número de *switches*), analisou-se o coeficiente de correlação intraclasse (ICC)^(24)^, utilizando o pacote “irr” para linguagem R^(24,25)^. Ressalta-se que considerou-se ICCs maiores ou iguais a 0,75 como correlações excelentes^(24)^. Foram obtidos os seguintes valores de ICC: 0,982 para o número de *clusters*, 0,958 para tamanho médio dos clusters, e 0,992 para o número de *switches*.

Etapa 6: Nova categorização dos itens discordantes

Após a análise de fidedignidade das análises através da concordância entre juízes, optou-se por reanalisar um a um os itens em que houve discordância entre os dois juízes. Para isso, dois novos juízes treinados categorizaram esses itens de forma independente. As pontuações dos dois novos juízes foram utilizadas para a tomada de decisão sobre a pontuação final desses itens.

Etapa 7: Versão final da categorização das variáveis

Considerando todas estas etapas, ao final foram definidas e tabuladas para análise todas as pontuações finais de cada participante nas quatro variáveis de análise (número total de palavras corretas evocadas, número de *clusters*, média do tamanho dos *clusters* e número de *switches*).

### Análise dos dados

Variáveis categóricas foram descritas em frequência absoluta e relativa enquanto variáveis contínuas foram descritas em média e desvio padrão. As características descritivas dos grupos foram comparadas utilizando o teste t de Student e Qui-Quadrado. Os desfechos do estudo (número total de palavras corretas evocadas, número de *clusters*, média do tamanho dos *clusters* e número de *switches*) foram comparados entre os grupos através do teste ANCOVA, utilizando idade e escolaridade como covariantes. Para estas essas análises, foi utilizado o *Statistical Package for Social Sciences* (SPSS) versão 25. Também foi realizada uma análise do coeficiente de correlação intraclasse (ICC) para inferir a concordância entre os juízes, utilizando o pacote “irr” para linguagem R^(25)^. O nível de significância utilizado neste estudo foi de 5%.

## RESULTADOS

A Tabela 1 apresenta os dados descritivos da amostra evidenciando diferenças significativas entre o GC e o GDA quanto à idade e escolaridade. GDA apresentava média de idade superior ao GC, enquanto este último apresentava média de escolaridade superior ao primeiro.

**Tabela 1:** Descrição da amostra, por grupo

	GC (n=39)	GDA (n=29)	P GC X GDA
Idade - média (DP)	71,5 (6,3)	78,6 (6,7)	<0,01*
Escolaridade - média (DP)	7,1 (3,4)	3,8 (3,1)	<0,01*
Sexo F - n (%)	34 (87,2)	20 (69)	0,07

* p≤ 0,05, teste ANCOVA

**Legenda:** DP = Desvio Padrão; F = Feminino; GC = Grupo Controle; GDA = Grupo Doença de Alzheimer

Os participantes do GC e do GDA foram então comparados quanto aos desfechos de interesse no estudo (número total de palavras corretas evocadas, número de *clusters,* média do tamanho dos *clusters* e número de *switches*), controlando as diferenças de idade e escolaridade, que foram incluídas como covariantes na análise estatística. A Tabela 2 apresenta os resultados das comparações demonstrando que a média do número de *switches* e do total de verbos gerados foi estatisticamente inferior no GDA.

**Tabela 2:** Desempenho na tarefa de fluência verbal de verbos por grupo

	GC (n=39)	GDA (n=29)	p
Número de *clusters* - média estimada (DP)	2,4 (0,2)	2,1 (0,3)	0,40
Tamanhos dos *clusters* - média estimada (DP)	1,6 (0,1)	1,5 (0,2)	0,60
Número de *switches* - média estimada (DP)	5,5 (0,5)	3,9 (0,6)	0,05*
Total de verbos gerados - média estimada (DP)	9,6 (0,6)	7, 0 (0,7)	0,01*

* p≤ 0,05, teste ANCOVA

**Legenda:** DP = Desvio Padrão; GC = Grupo Controle; GDA = Grupo Doença de Alzheimer

Adicionalmente, foi realizada a comparação dos desfechos do estudo apenas dentro do GDA comparando os participantes com demência leve com aqueles com demência moderada. Não houve diferença estatisticamente significativa para nenhuma das variáveis analisadas (Tabela 3).

**Tabela 3:** Comparações dos desfechos do estudo entre indivíduos com DA leve e DA moderada

	DA leve (n=18)	DA moderada (n=11)	p
Número de *clusters* - média estimada (DP)	2,1 (0,3)	1,3 (0,4)	0,15
Tamanhos dos *clusters* - média estimada (DP)	1,7 (0,2)	1,3 (0,3)	0,28
Número de *switches* - média estimada (DP)	3,5 (0,6)	2,4 (0,7)	0,24
Total de verbos gerados - média estimada (DP)	7,0 (0,7)	5,0 (0,9)	0,09

**Legenda:** DA = Doença de Alzheimer; DP = Desvio Padrão

## DISCUSSÃO

Esse estudo teve por objetivo investigar o desempenho na tarefa de fluência de verbos de pessoas com DA em comparação à idosos cognitivamente saudáveis. Para isso, foi realizado um minucioso processo metodológico para a categorização dos verbos de acordo com critério semântico, para então serem extraídas as variáveis: número total de palavras corretas evocadas, número de *clusters*, média do tamanho dos *clusters* e número de *switches*. Para a categorização dos verbos, o presente estudo baseou-se na classificação de Smirnova et al.^(22)^, classificação também utilizada em um estudo prévio com uma amostra de idosos com DA e saudáveis^(23)^. Entretanto, a organização exposta aqui diferiu um pouco da classificação de Smirnova et al.^(22)^, pois foram criadas subcategorias dentro das duas grandes categorias propostas por estes autores. Essa decisão ocorreu ao observar os verbos gerados pelos nossos participantes e as estratégias semânticas empregadas por eles, que demonstraram a necessidade de uma classificação mais refinada das categorias de verbos, a fim de não perder a riqueza de informações fornecida por nossa amostra.

Quanto à comparação dos desfechos de interesse entre o GC e o GDA, os dados sugerem que há uma diferença quantitativa na produção de verbos entre os grupos, demonstrando uma produção menor de palavras entre os sujeitos com DA em comparação ao grupo controle, como já descrito em outros estudos^(11,13,14)^. A classe dos verbos é considerada a que mais exige uma complexa organização semântica e seria, portanto, a mais sensível a déficits.

Neste estudo, os grupos diferiram com ao número de *switches*, mas não quanto ao número e tamanho dos *clusters*. Isso vai ao encontro dos achados de outros estudos que compararam o desempenho na fluência verbal semântica de idosos saudáveis e com DA quanto às variáveis de *clustering* e *switching* ^(10,26,27)^. Um número reduzido de *switches* sugere que os indivíduos com DA usam estratégias menos diversificadas para evocar os verbos e, possivelmente, apresentam como consequência um número reduzido no total de verbos evocados. Deste modo, este prejuízo específico na tarefa de fluência de verbos seria decorrente de déficits no funcionamento executivo e não necessariamente devido a dificuldades de acesso léxico-semântico desta classe de palavras (ou pelo menos as dificuldades executivas nesta tarefa seriam predominantes às dificuldades léxico-semânticas).

O pior desempenho no *switching* de verbos de sujeitos com DA em comparação a idosos sem DA, talvez decorra de dificuldades relacionadas à memória de trabalho. Um estudo prévio investigou a relação da performance da fluência verbal com o desempenho da memória de trabalho em idosos com e sem declínio cognitivo utilizando a metodologia de *clustering* e *switching* ^(4)^. O decréscimo nas habilidades de *switching* foi relacionado com o declínio da memória de trabalho. A análise do *switching* utilizada na fluência verbal se mostrou sensível às possíveis alterações nas habilidades relacionadas às funções executivas também em um estudo com adultos maiores de 50 anos com doença neurodegenerativa^(13)^.

Outra hipótese seria de que os déficits na fluência de verbos tenham relação com déficits de linguagem. Um estudo comparou o desempenho de idosos saudáveis, idosos com DA leve e idosos com DA moderada em tarefas de fluência de verbos e de nomeação de verbos^(12)^. Nela, concluíram que os pacientes com DA apresentaram tanto prejuízo na quantidade de palavras evocadas na fluência de verbos, quanto na nomeação dos verbos^(12)^. Entretanto, quando comparados os grupos quanto a gravidade da DA, encontraram que os com a doença mais avançada obtiveram pior desempenho na tarefa de nomeação, mas não diferiram quanto à fluência de verbos, da mesma forma que ocorreu neste estudo^(12)^. Tal estudo sugere que a dificuldade dos sujeitos com DA em processar verbos tem um predomínio semântico, o que não exclui a influência de prejuízos em outros domínios cognitivos^(12)^. Como o desempenho no processamento de verbos também depende da tarefa utilizada para a emissão de verbos, podemos concluir com base nos resultados da presente pesquisa que, na tarefa de fluência de verbos, o déficit executivo é o que mais dirige o desempenho dos sujeitos, demonstrando a maior sensibilidade desta tarefa ao funcionamento executivo do que ao processamento semântico dos verbos.

Adicionalmente, mesmo não tendo sido o foco do estudo, foram observadas algumas diferenças qualitativas na produção de verbos dos participantes deste estudo. De uma forma geral, os participantes do grupo com DA utilizaram palavras complementares ao descrever verbos, isto parece estar diretamente ligado a qualidade do verbo, sendo evocados verbos que podem ser considerados mais generalistas e que podem ser utilizados em contextos menos específicos (por exemplo: “fazer comida”, “fazer compras”). Isso pode sugerir uma dificuldade de evocação de verbos menos frequentes na língua portuguesa. Também pode haver uma dificuldade na compreensão da tarefa em si, que pode ter ocorrido no momento de compreender a instrução ou no processo de manter a instrução em mente, o que sugere um declínio na memória operacional. No entanto, os dados qualitativos obtidos neste estudo são insuficientes para o aprofundamento destas questões, se mostrando como achados secundários da pesquisa. Tal fenômeno poderá ser explorado com mais ênfase em estudos futuros.

A avaliação dos *clusters* levou em conta as palavras repetidas, isto difere da avaliação mais usual dos dados obtidos nas tarefas de fluências, que considera apenas o número total das palavras não repetidas. Esta decisão é baseada no pressuposto de que a análise dos agrupamentos ajuda a verificar as estratégias de evocação utilizadas pelos participantes. Os dados obtidos por este estudo sugerem que os pacientes com DA utilizam menos estratégias semânticas, evocando verbos sem conexão aparente entre si, quando comparados ao grupo controle. Ainda que haja uma correlação evidente entre o número de palavras evocadas e o número de agrupamentos, a primeira medida perde a capacidade explicativa deste nível de análise.

Finalmente, este estudo apresenta dados que demonstram que o desempenho na fluência de verbos, quanto ao número total de verbos evocados e a capacidade de *switching,* está prejudicada em pessoas com DA. Especificamente nesta tarefa de fluência de verbos, a dificuldade pode ter uma natureza predominantemente executiva, demonstrando a dificuldade dos sujeitos em utilizar estratégias cognitivas para evocar verbos. Estes achados podem ser úteis em diferentes aspectos. Primeiro, é possível que a tarefa de fluência de verbos e a habilidade de *switching* nesta tarefa sejam marcadores de conversão da DA, mas não necessariamente de progressão da doença (pois não houve diferença significativa dos desfechos estudados entre pessoas com DA leve e moderada). Segundo, esta característica cognitiva é relevante de ser considerada no planejamento terapêutico da reabilitação neuropsicológica. Por exemplo, em intervenções que visam a redução de anomias, o treinamento do uso de diferentes estratégias para evocar verbos/ações pode auxiliar os pacientes a acessarem essas palavras com mais facilidade. Terceiro, os achados sugerem que os lobos temporais (atingidos já nas fases iniciais da DA) e suas conexões também são importantes na busca por estratégias cognitivas para evocar verbos.

Sugerimos que este tema de pesquisa seja mais profundamente explorado em estudos futuros com amostras maiores e que investiguem também as diversas apresentações da DA (Ex. perfis amnéstico, disexecutivo), já que isso não foi possível na presente pesquisa. A metodologia empregada aqui também pode ser utilizada em populações com outras doenças neurológicas ou mesmo em sujeitos saudáveis de diferentes faixas etárias e níveis de escolaridade, a fim de compreender de forma mais profunda o processamento neural dos verbos e a utilidade da tarefa de fluência de verbos.

## CONCLUSÃO

Este estudo demonstrou que sujeitos com DA tem um prejuízo no número de verbos gerados corretamente na tarefa de fluência de verbos e na habilidade de *switching* quando comparados a idoso saudáveis. Os resultados demonstram que a fluência de verbos é mais sensível aos déficits cognitivos decorrentes de disfunção executiva que ocorrem na população estudada, do que aos déficits semânticos.

É possível que a tarefa de fluência de verbos seja empregada na rotina clínica para a avaliação cognitiva desta população auxiliando no diagnóstico, acompanhamento e na tomada de decisão para a reabilitação neuropsicológica, através também das medidas de *switching e clustering*. Ainda, no âmbito científico, a metodologia empregada no presente estudo pode ser mais explorada em outras doenças neurológicas para compreensão de seus perfis cognitivos.
